# The Eyes

**Published:** 1893-01

**Authors:** 


### THE EYES.

The keenness of the sailor's organs of sight is almost proverbial. This effect has two causes. The cold salt spray dashing into the seaman's eyes strengthens and hardens them. Also, the mariner's practice of constantly piercing the atmosphere to see something, often absolutely undiscernible, greatly trains the organ in clever acuteness. A thought is immediately suggested. Would it not be beneficial to teach the children to test their ability to see distant objects? The hands of the court-house clock, an incoming vessel, a faintly appearing train, the rapidly fading forms of birds in flight, and many other objects that the little ones would be eager to notice if so directed, would aid to expand and perfect the various delicate and minutely beautiful parts which compose the eye.

Infants are frequently born with eyes so weak that they "water" upon exposure to wind or light, even when judiciously advanced to these. This weakness may be cured by frequent bathing with water of the saltness and temperature of tears, or, as in my experience has been of more value, dashing cold water over the eyes each time before being taken out, and never bathing the baby's face, especially about the eyes with warm water. Cold tea is also recommended, and may do the work for some and fail in other cases.

